# Transvesical Versus Posterior Approach to Retzius-Sparing Robot-Assisted Radical Prostatectomy: A Retrospective Comparison With a 12-Month Follow-Up

**DOI:** 10.3389/fonc.2021.641887

**Published:** 2021-04-15

**Authors:** Wen Deng, Cheng Zhang, Hao Jiang, Yulei Li, Ke Zhu, Xiaoqiang Liu, Luyao Chen, Weipeng Liu, Ju Guo, Xiaochen Zhou, Bin Fu, Gongxian Wang

**Affiliations:** ^1^ Department of Urology, the First Affiliated Hospital of Nanchang University, Nanchang, China; ^2^ Jiangxi Institute of Urology, Nanchang, China

**Keywords:** prostate cancer, robot-assisted radical prostatectomy, Retzius-sparing, transvesical approach, posterior approach

## Abstract

**Objectives:**

To assess the perioperative, functional, and oncological outcomes of transvesical robot-assisted radical prostatectomy (T-RARP) and posterior robot-assisted radical prostatectomy (P-RARP) for localized prostate cancer.

**Materials and Methods:**

We analyzed the data of 96 patients who underwent T-RARP or P-RARP for localized prostate cancer between January 2017 and June 2019 in a retrospective fashion.

**Results:**

No significant differences in the baseline characteristics existed between the T-RARP and P-RARP arms. Both interventions were successfully performed without open conversion in either group. T-RARP was associated with a slightly more operative time (135.3 vs. 127.3 min) and estimated blood loss (105.2 vs. 94.2 mL) than P-RARP, but the differences were not significant (both *p* > 0.05). The likelihood of transfusion, ≤Grade II, and >Grade II postoperative complications, pT3a disease and positive surgical margins in the T-RARP group was comparable with that in the P-RARP group. No significant differences were noted between these two arms in terms of UC at the removal of catheter and nocturia (*p* = 0.750 and *p* = 0.684, respectively), and all included patients recovered UC at 3 months postoperatively. The median International Index of Erectile Function-5 score in both groups remains comparable before and after RARP. The patients in the T-RARP and P-RARP groups had a similar biochemical recurrence-free survival (*p* = 0.387).

**Conclusions:**

Both T-RARP and P-RARP by experienced hands are feasible for well-selected patients with prostate cancer, obtaining similar outcomes in terms of perioperative results, UC and erectile function, and oncological control within short-term follow-up.

## Introduction

Prostate cancer, the second most frequent cancer and the fifth leading cause of cancer-specific mortality among men worldwide (1), is increasingly being detected with serum prostate-specific antigen (PSA) screening, especially the localized ones (2). In randomized clinical trials, radical prostatectomy (RP) showed an advantage in overall and cancer-specific survival benefits over watchful waiting when managing localized prostate cancer (3–5). With the executive facilitation of robotic surgery in crucial surgical procedures for RP, including bladder neck preservation, nerve-sparing dissection, and prostate apex management, robot-assisted RP (RARP) has been widely disseminated and rapidly become the most common surgical approach for treating localized prostate cancer since 2001 (6, 7).

However, technical improvements and a more thorough understanding of the anatomy of the prostate and its surrounding structures did not help more in further limitations in the incidence of urinary incontinence (8, 9), a particularly feared complication of RP (9, 10). This situation was considerably improved following the introduction of an innovative surgical method, well known as the “Retzius-sparing” approach or the posterior approach, by Bocciardi et al. (11) The posterior method merely approaches the prostate gland through Douglas’ pouch, thus circumventing the related anatomical structures in Retzius’ space, which are strongly related to urinary continence (UC) (11). The advantage of the posterior approach over the conventional approach in the early recovery of UC has been repeatedly confirmed with high-level evidence from several well-designed prospective randomized trials (9, 10, 12).

The transvesical approach to RARP was initially applied by Desai et al. (13) on two cadavers employing the da Vinci-S robotic system, proving this method executable. Given the absence of disruption to Retzius’ space by posterior and transvesical approaches, we hypothesized that transvesical RARP (T-RARP) could yield similar functional outcomes to posterior RARP (P-RARP) in well-selected patients. Our team firstly applied this approach on such patients harboring low-risk localized prostate cancer with several appropriate modifications made and detailedly described the whole process for T-RARP on a multi-port basis with the da-Vinci Si/Xi system (14).

The present paper was the first study retrospectively designed to compare the impact of the transvesical and posterior approaches to Retzius-sparing RARP on perioperative outcomes, recovery of UC and erectile function, and oncologic control within 12-month follow-up.

## Materials and Methods

### Data Source and Ethics Statement

All demographic, clinical, and pathological information was retrospectively obtained from our prospectively maintained database after acquiring the approval of the Ethics Committee of the First Affiliated Hospital of Nanchang University. Written informed consent was obtained from all these patients.

### Patients Selection

Between January 2017 and June 2019, all patients bearing primary localized prostate cancer were screened out and enrolled for the final analysis according to the following eligibility criteria (1): total PSA < 20 ng/mL, (2) Gleason score ≤ 7, (3) clinical stage T1-T2cN0M0, (4) prostate volume < 80 mL, and (5) surgical treatment with P-RARP or T-RARP. Only when all these criteria were simultaneously met were patients included, and the others were excluded from our analysis. Finally, 96 patients fulfilling the inclusion criteria were analyzed in the study. Among them, 44 and 52 patients were stratified by surgical approach to the transvesical and posterior arms, respectively. No patient had a history of abdominal surgery. All patients underwent preoperative prostate magnetic resonance imaging and bone scintigraphy.

### Technical Considerations

P-RARP was performed by two highly experienced surgeons (Wang GX and Fu B) in compliance with the techniques established by Bocciardi et al. (11), while T-RARP was completed by Wang GX since January 2018 following the surgical steps described in our published study (14). These two surgeons (Wang GX and Fu B)had received standardized training in robotic surgery and performed more than 300 robot-assisted radical prostatectomies *via* the anterior or posterior approaches by the time that the transvesical approach was applied on the first patient. The patients in the P-RARP group were usually assigned at the discretion of the highly experienced surgeons according to tumor and patient characteristics, while those in the T-RARP group were discretionarily enrolled after elaborative descriptions of why and how to conduct T-RARP, the differences between the various approaches to preforming RARP and alternative choices of disease management and following the acquisition of written informed consent including all information mentioned above. Extended pelvic lymph node dissection (ePLND) was performed if the preoperative estimated risk for nodal involvement exceeded 5%, while nodal dissection could be omitted at a low risk of missing positive nodes in all other cases. All surgeries in both groups were conducted following the nerve-sparing technique.

Similar to the posterior approach, the transvesical approach obviates any disruptions to Retzius’s space. Unlike posterior RARP (P-RARP), the prostate and periprostatic structures could be exposed directly in-line by applying the transvesical approach, thus translating into easier anterior or lateral dissection. In addition, urethrovesical anastomosis was performed similarly to the transperitoneal anterior technique. All these factors could result in a shorter learning curve. The transvesical approach was completely confined to the deep bony pelvis without requiring a steep Trendelenburg position or any bowel retraction.

### Variable Definition and Endpoints

For each patient, the preoperative baseline characteristics, including age, body mass index (BMI), diabetes mellitus, hypertension, preoperative serum total PSA, prostate volume on the basis of transrectal ultrasound, clinical TNM stage, preoperative erectile function quantified in accordance with the International Index of Erectile Function (IIEF)-5 score (15), and biopsy Gleason score, were retrieved from our database.

The perioperative outcomes included operative time (OT), estimated blood loss (EBL), open conversion, transfusion, postoperative complications (which were classified and graded according to the modified Clavien–Dindo system) (16), urethral catheterization length, and hospital stay length. Pathologic outcomes included pathological staging, positive surgical margin (PSM), and specimen Gleason score. Information about complications was collected by doctors *via* chart reviews or face-to-face interviews every 3 months when patients underwent regular postoperative re-evaluation, while attempts were made to obtain missing data *via* telephone interview.

The median follow-up for both the transvesical and posterior groups was 12 months. PSA tests were conducted for each patient every 3 months within the postoperative 12 months to monitor biochemical recurrence (BCR), which was defined as a PSA level >0.2 ng/mL on two consecutive measurements. Information regarding UC, which was perceived as requiring no pad or preventively using one dry pad per day, at removal of catheter and postoperative 3 months and 12 months after surgery, was also compared. Erectile function recovery was defined as a IIEF score ≥ 22.

### Statistical Analysis

Continuous data in a normally distributed fashion were analyzed using the independent t-test and presented as the mean and standard deviation, while those in a non-normal distribution were calculated using the Wilcoxon rank–sum test and presented as the median and interquartile range. Dichotomous variables were compared using the Pearson chi-squared test or Fisher’s exact test. The Kaplan–Meier method performed with log-rank test was employed to estimate BCR-free survival and function recovery on STATA version 12.0 (STATA corp., College Station, TX). All other statistical analyses were conducted utilizing the SPSS software package (version 22, IBM SPSS Statistics, Armonk, NY), and results with a two-sided *p*-value < 0.05 were considered statistically significant.

## Results

During the study period, a total of 96 eligible and consenting patients were grouped into the T-RARP (n = 44) or P-RARP (n = 52) arm according to the surgical approach. As summarized in **Table 1**, there were no statistically significant differences between the two groups in terms of mean age, BMI, preoperative total PSA, or prostate volume (*p* > 0.05). 3 (6.8%) and 5 (9.6%) patients had a prostate volume between 50 and 80 ml in the T-RARP and P-RARP groups, respectively (*p* = 0.723). The patients in the T-RARP group had a slightly higher proportion of diabetes mellitus (15.9% vs. 13.5%, *p* = 0.735) and a lower but not significantly different proportion of hypertension (40.9% vs. 44.2%, *p* = 0.743) than those in the P-RARP group. The rate of clinical T2c tumors in the P-RARP group was slightly higher than that in the T-RARP group (9.6% vs. 0%), without a significant difference (*p* = 0.090). The median preoperative IIEF-5 score and biopsy Gleason score were also statistically similar between the two groups. 14 (31.8%) and 24 (46.2%) patients bore prostate cancers with biopsy Gleason scores of 7 in the T-RARP and P-RARP groups, respectively (*p* = 0.152).

**Table 1:** Preoperative baseline characteristics.

	Transvesical approach (*n* = 44)	Posteriorapproach (*n* = 52)	p
Age, years, mean (SD)	64.8 (6.5)	67.4 (7.4)	0.081
BMI, kg/m2, mean (SD)	26.3 (3.2)	27.2 (3.6)	0.208
Diabetes mellitus (yes), n (%)	7 (15.9%)	7 (13.5%)	0.735
Hypertension (yes), n (%)	18 (40.9%)	23 (44.2%)	0.743
Preoperative serum total PSA, ng/mL, mean (SD)	13.3 (3.7)	15.2 (7.4)	0.097
Prostate volume, mL, mean (SD)	35.5 (10.2)	38.6 (9.3)	0.125
Prostate volume ≥ 50 and < 80 ml, n (%)	3 (6.8%)	5 (9.6%)	0.723
Preoperative IIEF-5 score, median (IQR)	17 (14, 20)	17 (14.25, 19)	0.402
cTNM stage, n (%)			0.090
cT1c	6 (13.6%)	12 (23.1%)	
cT2a	29 (65.9%)	27 (51.9%)	
cT2b	9 (20.5%)	8 (15.4%)	
cT2c	0 (0%)	5 (9.6%)	
Biopsy Gleason score, median (IQR)	6 (5, 7)	6 (5, 7)	0.301
Biopsy Gleason score = 7, n (%)	14 (31.8%)	24 (46.2%)	0.152

BMI, body mass index; IIEF, International Index of Erectile Function; SD, standard deviation; IQR, interquartile range.

The perioperative outcomes are reported in **Table 2**. The T-RARP group was correlated with a slightly longer mean OT (135.3 vs. 127.3 min, *p* = 0.159) and higher mean EBL (105.2 vs. 94.2 mL, *p* = 0.361) than the P-RARP group. 3 (6.8%) and 5 (9.6%) patients underwent ePLNDs in the T-RARP and P-RARP arms, respectively (*p* = 0.723), and no cases with positive lymph nodes were found in either group. The overall degree of open conversion and transfusion were comparable between the two arms. The median hospital stay length in the T-RARP group was statistically similar to that in the P-RARP group (7 vs. 7 days, *p* = 0.852).

**Table 2:** Comparison of perioperative outcomes.

	Transvesical approach (*n* = 44)	Posteriorapproach (*n* = 52)	p
Operative time, min, mean (SD)	135.3 (26.2)	127.3 (28.8)	0.159
Estimated blood loss, mL, mean (SD)	105.2 (63.6)	94.2 (53.7)	0.361
Open conversion, n (%)	0 (0%)	0 (0%)	–
Transfusion, n (%)	0 (0%)	2 (%)	0.498
ePLND, n (%)	3 (6.8%)	5 (9.6%)	0.723
Postoperative pathology			
Pathological T stage, n (%)			0.417
pT2a	26	29	
pT2b	14	12	
pT2c	3	10	
pT3a	1	4	
Positive lymph node, n (%)	0 (0%)	0 (0%)	–
Specimen Gleason score, median (IQR)	6 (5, 7)	6 (5, 7)	0.360
Positive surgical margin, n (%)	5 (11.4%)	7 (13.5%)	0.757
Urethral catheterization, days	7	7	–
Hospital stay, days, median (IQR)	8 (7, 9)	8 (7, 8)	0.852

SD, standard deviation; IQR, interquartile range; ePLND, extended pelvic lymph node dissection.

Oncologically, the proportions of pT3a disease and PSM in the P-RARP group were slightly higher than those in the T-RARP group (3.8% vs. 0% and 17.3% vs. 15.9%, respectively), but were not significantly different (p = 0.498 and p = 0.855, respectively). The pathological data regarding the median specimen Gleason score in the two arms were also similar (6 vs. 6, p = 0.360).

The statistical comparability remains in terms of the incidence of low-grade (≤Grade II) and high-grade (>Grade II) complications (**Table 3**). All low-grade complications were successfully managed with conservative treatment, while one case of symptomatic lymphocele in the P-RARP arm required percutaneous drainage during the study period.

**Table 3:** Comparison of postoperative complications.

	Transvesical approach (*n* = 44)	Posterior approach (*n* = 52)	p
≤ Grade II complications, n (%)	3 (6.8%)	5 (9.6%)	0.723
Asymptomatic urinary infection	2 (4.5%)	2 (3.8%)	
Nocturia	1 (2.3%)	2 (3.8%)	
Fever	0 (0%)	1 (1.9%)	
Dysuria	0 (0%)	0 (0%)	
> Grade II complications, n (%)	0 (0%)	1 (1.9%)	1.000
Symptomatic lymphocele	0 (0%)	1 (1.9%)	

**Figure 1:**
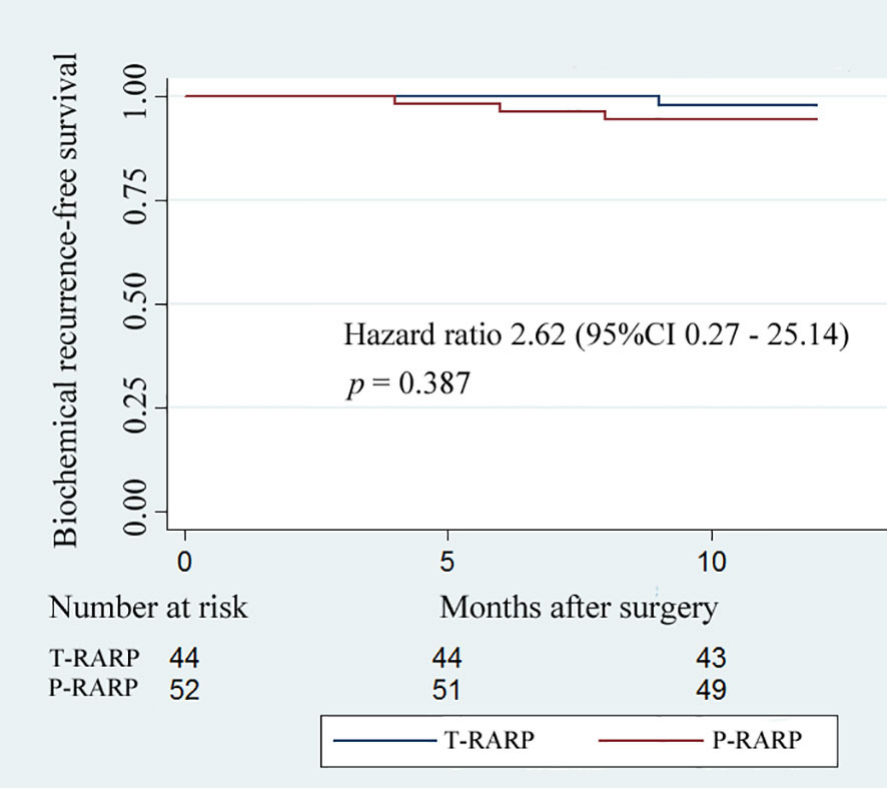
Kaplan–Meier curves showing biochemical recurrence−free survival for patients undergoing the transvesical and posterior approaches to robot-assisted radical prostatectomy within postoperative 12-months follow-up.

The data with respect to the mean total PSA at postoperative 1 week and 12 months revealed a similar outcome for the two groups (**Table 4**). During the postoperative12-month follow-up, the occurrence of BCR in the T-RARP and P-RARP groups was exhibited on one and three patients, respectively. The *p*-value for the difference in BCR-free survival between the two groups was 0.387 (hazard ratio: 2.62, 95% CI: 0.27 - 25.14) (**Figure 1**).

**Table 4:** Comparison of surgical outcomes.

	Transvesical approach (*n* = 44)	Posterior approach (*n* = 52)	p
Oncology: postoperative total PSA, ng/mL, mean (SD)			
Postoperative 1 week	2.133 (1.914)	2.358 (1.537)	0.526
Postoperative 12 months	0.046 (0.029)	0.051 (0.025)	0.308
Urinary continence			
Continent on removal of catheter, n (%)	40 (90.9%)	46 (88.5%)	0.750
Continent at postoperative 3 months, n (%)	44 (100%)	52 (100%)	–
Continent at postoperative 12 months, n (%)	44 (100%)	52 (100%)	–
Erectile function, median (IQR)			
IIEF-5 score at postoperative 3 months	15 (10, 18)	14 (9, 17)	0.431
IIEF-5 score at postoperative 12 months	14 (10, 16)	13 (9,15)	0.458

SD, standard deviation; IQR, interquartile range.

**Figure 2:**
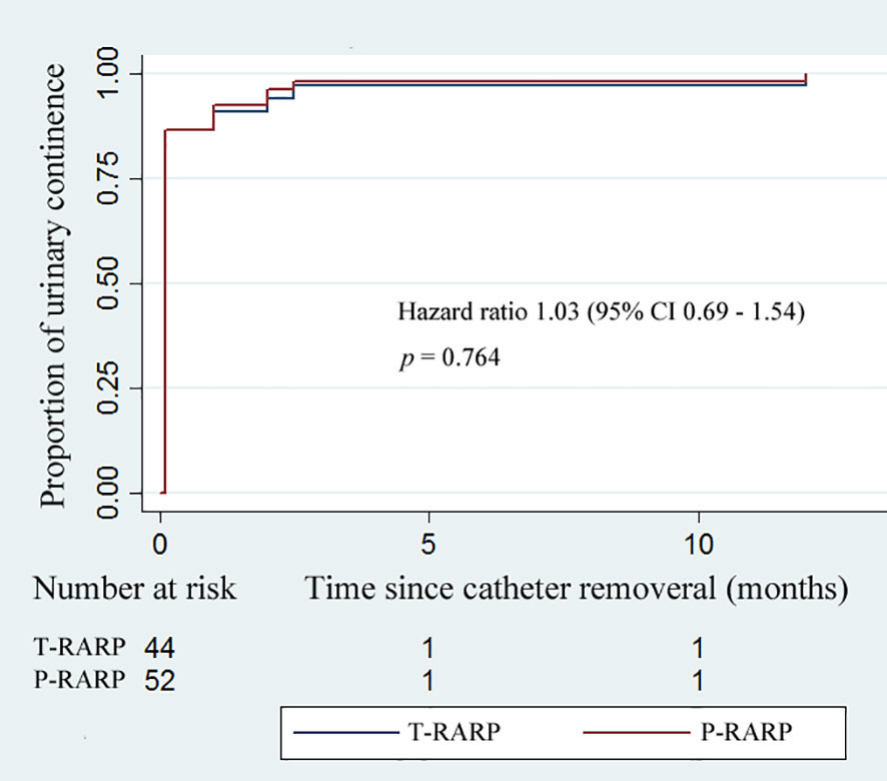
Kaplan–Meier curves showing the proportion of urinary continence (UC) in patients undergoing the transvesical and posterior approaches to robot-assisted radical prostatectomy within postoperative 12-months follow-up. UC was defined as requiring no pad or preventively using one dry pad per day.

The Foley catheter was removed routinely on postoperative day 7. As shown in **Table 4**, 40/44 patients (90.9%) in the T-RARP group and 46/52 patients (88.5%) in the P-RARP group achieved immediate UC (*p* = 0.750). Within 1 month postoperatively, one and two patients complained of nocturia after discharge in the T-RARP and P-RARP arms, respectively, and all symptomatic complaints were in a gradual remission with solifenacin succinate. Other patients gradually disengaged from urinary incontinence without medical intervention. No patient complained of symptoms related to bladder outlet obstruction within 12 months postoperatively. All patients included in this analysis recovered to UC by postoperative 3 months. The p-value of the difference in the proportion of urinary continence was 0.764 between the two arms (hazard ratio: 1.03, 95% CI: 0.69 - 1.54) (**Figure 2**).

**Figure 3:**
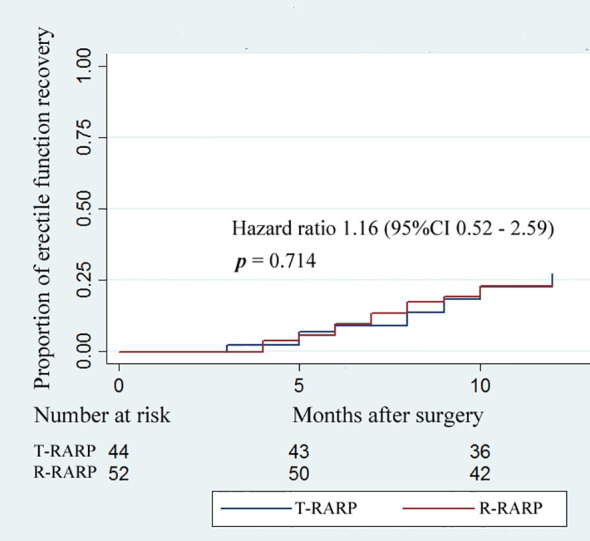
Kaplan–Meier curves showing the proportion of postoperative erectile function recovery according to surgical type within postoperative 12-months follow-up. Erectile function recovery was defined as a IIEF score ≥ 22.

Within the follow-up period, no significant difference was found in the median IIEF-5 score at postoperative 3 months and 12 months between the T-RARP and P-RARP arms (15 vs. 14, p = 0.431; 14 vs. 13, p = 0.458), as presented in **Table 4**. There was no significant difference in proportion of postoperative erectile function recovery between the T-RARP and P-RARP groups (p = 0.714) (**Figure 3**).

## Discussion

The goal of contemporary RP surgery is to minimize its impact on patients’ quality of life and function without compromising oncological control (17, 18). The development of postprostatectomy incontinence (PPI) is intricately blamed on multiple pathophysiologic mechanisms (8, 10). In addition to biological/preoperative factors, such as patient age, pre-existing lower urinary tract symptoms, and high BMI, anatomic support and pelvic innervation are also regarded as important factors in the etiology of PPI (8). Hence, several creative methods have been consequently employed to improve the rates of immediate and long-term UC, such as bladder neck preservation (19), posterior reconstruction (20), and sling suspension (21). Unlike UC, erectile function is in a clear anatomical and physiological relation to the periprostatic neurovascular bundles (22). Nerve-sparing RP has been recommended as the approach of choice for all men with normal erectile function and organ-confined disease (23).

Given the magnifications and the millimetric robotic instruments used during RARP, the detailed understanding of the periprostatic anatomy has been greatly improved (24), thus translating into a more precise dissection and a higher level of tissue preservation during prostatectomy (25). On the strength of more detailed knowledge of the periprostatic anatomy obtained with robots, Desai et al. (13) tentatively applied the transvesical approach to RARP on two fresh cadavers, demonstrating the technical feasibility of the procedure in the human body. Gao et al. (26) conducted single-port transvesical laparoscopic RP in 16 consecutive patients with organ-confined prostate cancer and concluded that the method is oncologically safe and technically feasible with promising functional outcomes regarding continence and potency. These previously reported knowledge and skilled experience in performing the anterior/posterior approach to RARP established a foundation for the implementation of transvesical RARP with multiple ports. Here, we conducted a retrospective study with the aim of comparing this novel surgical approach with the posterior approach in terms of perioperative, functional, and oncological outcomes. The data indicated that these two approaches are similar in terms of OT, EBL, proportions of postoperative complications and UC, erectile function preservation, and BCR-free survival.

The comparable OT, EBL, and rates of open conversion and transfusion between the two arms were somewhat surprising when comparing this novel approach with the maturely applied one. This may be explained *via* a retrospective review of the following key technical points. Smaller (< 60 ml) prostates are easily exposed when the cystotomy is expanded laterally using suspension stitches in advance. Similar to the posterior approach, the initial dissection starts posteriorly, thus greatly shortening the learning curve in a highly experienced hand. The process of urethrovesical anastomosis in this transvesical fashion is almost identical to that in the anterior approach, which we were proficient in (25). The insignificantly higher frequency (3.8%) of > Grade II postoperative complications in the P-RARP group may be related to the higher rate of cT2c prostate cancer. Notably, the published proportions of >Grade II postoperative complications after P-RARP range from 2.0% to 8.3% (6, 12), revealing the technical safety of the two approaches we adopted. We also compared the first five patients with the last one who underwent T-RARP in terms of the intraoperative outcomes, ending with insignificant differences in all these endpoints. Given that T-RARP procedure after bladder neck excision is greatly similar to the transperitoneal anterior technique which we are well experienced in, the learning curve of transvesical approach could be greatly shortened, especially in the highly experienced hand.

Regarding the similar recovery of UC and erectile function, these results could be attributed to the common advantage of these two Retzius-sparing surgeries for localized prostate cancer. Most importantly, the related anatomical structures in the retropubic space are protected from any destruction in the same way. The similar preservation of neurovascular structures during the initial proximity to the prostate from the posterior side could easily interpret the recovery of erectile function in both groups. The nerve-sparing technique was also helpful in recovering UC to a certain degree (22). In our study, all biological factors, including patient age and BMI, were comparable, which contributed to the similar functional recovery between the T-RARP and P-RARP groups. All incidences of urinary incontinence in both groups occurred and subsequently disappeared with medical intervention within the first 3 months. The highest possibility of urinary incontinence-related complaints has been reported to appear in the first 2–6 months postoperatively (27), while the published frequencies of UC at 12 months after P-RARP vary from 96% to 100% on the grounds of the definitions of incontinence, severity, discomfort and methodology of assessment (28–30). Our promising frequency of UC (100%) in either arm reached similar high levels, which were achieved in this published P-RARP series, reflecting the efficiency of P-RARP and T-RARP in functional protection for patients with localized prostate cancer.

Surgical innovations in the management of solid cancer should be tempered with a critical analysis of oncological control. It is worth noting that the tendency of PSM in the P-RARP (17.3%) arm was insignificantly higher than that in the T-RARP (15.9%) group in this analysis. The difference may be due to the higher rate of pT3a disease in the P-RARP arm, as the more extensive the cancer, the higher the risk of positive margins (31). Moreover, one may argue that the risk of PSM is related to surgical experience and RARP training (6, 31). The impact of this predictor was also limited by the surgeon’s high procedure volume and the technical similarity described before. PSM undoubtedly increases the risk of disease recurrence. However, the interval between PSM and the time to event is too long and depends mostly upon other variables (6, 31). Abdollah et al. (32) also found that PSMs by itself did not increase the risk of clinical recurrence in patients with organ-confined diseases or a Gleason score ≤ 7, which extremely approach the inclusion criteria of our study. Moreover, the prognostic significance of focal PSMs after RP remains to be elucidated. Many studies have noted that focal PSMs do not significantly affect BCR-free survival in patients with prostate cancer (33). Overall, the BCR-free survival tended to be similar in our two study arms. The postoperative fraction of patients with BCR (3/52) in the P-RARP group of our analysis was lower than that (13.3%) reported by Chang et al. (29), attributing to the smaller mean tumor size and proportion of pT3 disease.

Several limitations should be recognized in our study. Structural limits in collecting the information were inevitable due to the retrospective designed fashion of the study. We could not further estimate oncological survival and functional outcomes due to the short-term follow-up and limited sample size. Certain complications may be underrated, especially ≤ Grade II complications, despite the thorough scrutiny of the medical records.

Despite these limitations, this retrospective study was the first designed to assess the perioperative, functional, and oncological outcomes of the T-RARP and P-RARP methods for localized prostate cancer to date, and our conclusions are drew and strengthened on the basis of the comparability of all baseline characteristics between the two arms and implementation of a rigorous methodology.

## Conclusions

Both T-RARP and P-RARP by highly experienced hands tended to be feasible for selected patients with prostate cancer, yielding similar outcomes in terms of perioperative results, UC and erectile function preservation, and oncological control within short-term follow-up. Our conclusions need to be confirmed further with prospectively randomized trials with large sample sizes and sufficiently long follow-ups.

## Data Availability Statement

The raw data supporting the conclusions of this article will be made available by the authors, without undue reservation.

## Author Contributions

Conception and design: BF, GW, and XZ. Acquisition of data: WD, CZ, HJ, YL, KZ and JG. Analysis and interpretation of data: WD, XL, JG, and LC. Statistical analysis: WD. Manuscript writing: WD and WL. Manuscript editing: BF, GW, and XZ. All authors contributed to the article and approved the submitted version.

## Funding

This study was supported by the Natural Science Foundation of China (Grant No. 81760457).

## Conflict of Interest

The authors declare that the research was conducted in the absence of any commercial or financial relationships that could be construed as a potential conflict of interest.
